# Fatal Lower Respiratory Tract Infection Following a Dog Bite in a Cirrhotic Patient: A Case Report of Zoonotic Sepsis

**DOI:** 10.7759/cureus.100209

**Published:** 2025-12-27

**Authors:** Athanasia Aidonopoulou, Konstantinos Katsos, Dimitrios Vlachodimitropoulos, Artemis A Dona, Emmanouil I Sakelliadis

**Affiliations:** 1 Department of Forensic Medicine and Toxicology, National and Kapodistrian University of Athens School of Medicine, Athens, GRC

**Keywords:** autopsy, dog bite, forensic medicine, forensic pathology, greece, sepsis

## Abstract

We report the case of a 66-year-old woman with a history of alcohol abuse and cirrhosis, living in poor housing conditions. She was found dead in her residence and was reported to have sustained a dog bite to the left upper limb approximately 20 days before death. According to police reports, she neither sought medical attention nor received antibiotic therapy following the incident. The wound was neglected, and external examination at autopsy revealed signs of cellulitis of the affected limb.

A medico-legal postmortem examination was performed to determine the cause of death. The autopsy revealed a lower respiratory tract infection, determined to be secondary to the soft tissue infection originating from the untreated dog bite. Given the underlying immunosuppression associated with advanced cirrhosis, the disease progressed systemically, ultimately leading to sepsis and death. The findings support a fatal outcome due to complications of an untreated soft tissue infection in an immunocompromised host.

A broad spectrum of zoonotic pathogens has been implicated in both localized and severe systemic complications following animal bites, particularly among immunocompromised individuals.

## Introduction

Dog bites represent the most common type of animal bite worldwide and are associated with a significant risk of secondary infection, despite most injuries being minor and self-limited [1,2]. In the United States, millions of dog-bite incidents occur annually, a substantial proportion of which require medical evaluation [2]. Although severe outcomes are uncommon, dog bite-related infections may, in rare cases, progress to systemic illness and death [1,2].

Patients with chronic liver disease, particularly those with cirrhosis, are at increased risk of severe infections due to cirrhosis-associated immune dysfunction [2]. Even minor skin injuries, including animal bites, may act as portals of entry for pathogens and lead to rapidly progressive and life-threatening infections in this population [2]. While epidemiological data indicate that males and children are more frequently affected by dog bites, host-related factors are more predictive of clinical severity than demographic characteristics alone [1,3,4].

This case report describes a rare and fatal complication following a dog bite in a patient with underlying liver cirrhosis. By illustrating the rapid progression from a bite injury to overwhelming infection in a high-risk host, this case highlights the importance of early recognition, prompt intervention, and careful clinical and forensic evaluation [2,5]. The rarity of such presentations and their potential medicolegal implications underscore the value of this case for both clinical and forensic practice.

## Case presentation

A 66-year-old woman with an unknown medical history was found dead in a supine position on her bed by neighbors. She was known to reside in poor housing conditions and reportedly had a history of alcohol abuse. Approximately 20 days before death, she sustained a dog bite to the left upper limb. Due to the absence of medical records and the circumstances surrounding her death, a medico-legal autopsy was requested by law enforcement to determine the cause of death.

**Figure 1:**
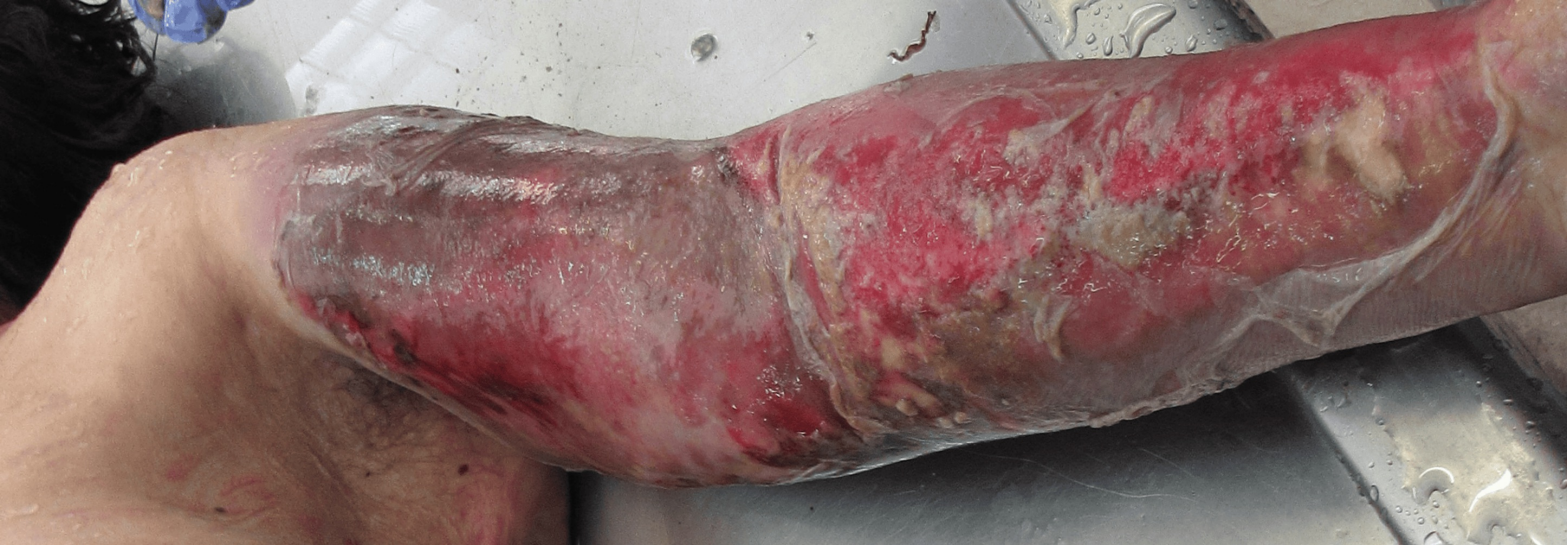
Left upper extremity Photo taken during the postmortem examination. Note the diffuse erythema and edema in the left upper extremity.

During external examination, diffuse erythema and edema of the left upper limb were noted, consistent with cellulitis, suggesting an ongoing soft tissue infection at the site of the previous dog bite (Figure 1).

**Figure 2:**
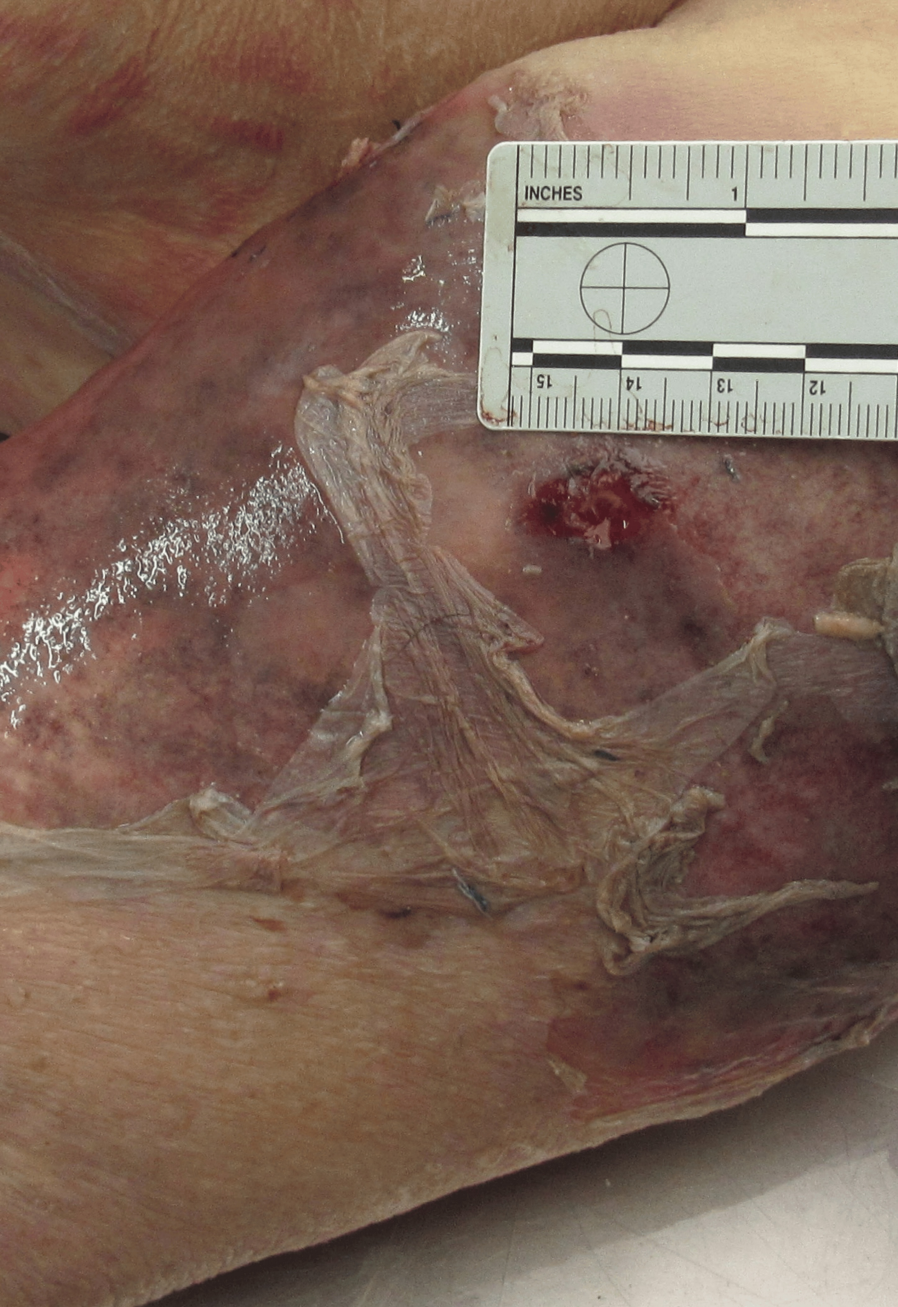
Wound on the left upper extremity. Photo taken during the postmortem examination. Left upper extremity close-up. Please note the wound on the left shoulder.

More specifically, a wound measuring approximately 1.5 x 1 cm was observed on the left shoulder (Figure 2).

Additionally, three bruises, each measuring approximately 1.5 cm in diameter, were identified on the left upper arm. The left forearm showed two abrasions of roughly 2 cm, while the left hand presented two abrasions, each measuring approximately 1 cm. The observed bruises were in the resolution stage, and both the wound and abrasions were crusted, indicating partial healing. Based on their appearance and healing characteristics, the estimated age of the injuries was consistent with the reported timeline of the dog bite incident, which occurred approximately 20 days before death.

Gross and histologic findings

**Figure 3:**
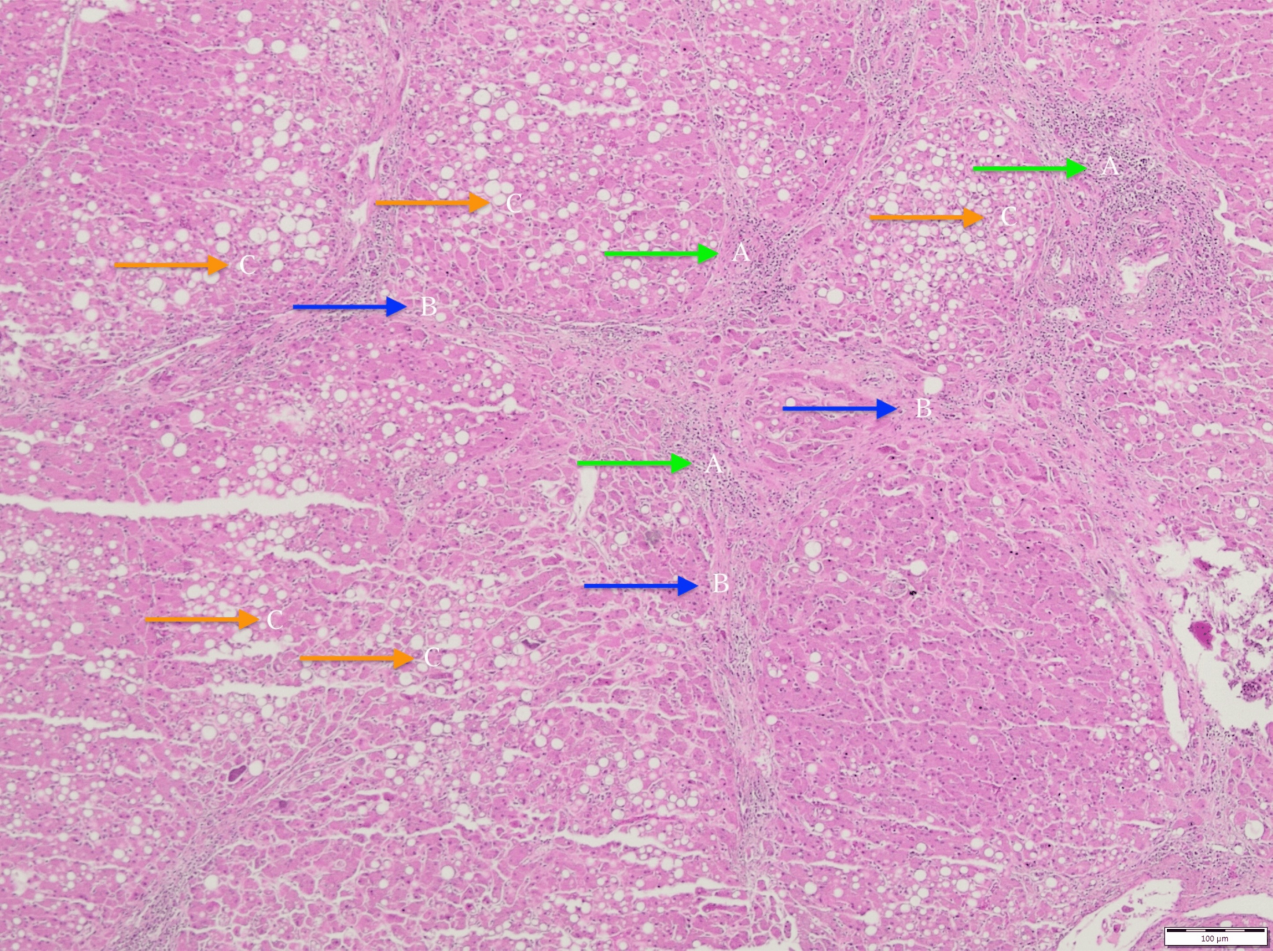
Histological analysis (Hematoxylin-Eosin x40) A: visible inflammatory infiltrations (blue arrows); B: fibrous bands, separating liver tissue (finding compatible with cirrhosis) (green arrows); and C: extensive steatosis of hepatocytes (orange arrows)

At autopsy, the liver showed a yellowish, greasy surface with multiple regenerative nodules interspersed with bands of bridging fibrosis, resulting in extensive distortion of the standard hepatic architecture. Histological analysis confirmed micronodular cirrhosis with features of steatohepatitis (Figure 3).

These morphological changes, in the context of a documented history of chronic alcohol consumption, were consistent with alcohol-related liver disease (ARLD).

The lungs demonstrated histologic features of lobar pneumonia in the red hepatization phase, characterized by alveolar dilation and intrabronchial mucus accumulation. Histological examination of the lungs confirmed pneumonia at a stage suggestive of secondary involvement, originating from a primary soft-tissue infection of the left upper limb sustained following a dog bite. The heart weighed 360 gms, and histopathological examination revealed mild coronary artery disease, along with extensive hypoxic injury to myocardial fibers, suggestive of ischemic stress likely related to impaired oxygenation. Examination of the spleen showed atrophy of the white pulp, indicative of alcohol-mediated splenic atrophy. Toxicological analysis revealed no evidence of illegal substances or prescribed medications at the time of death.

The autopsy findings support a clinical course in which a lower respiratory tract infection developed after the initial soft tissue injury. In the context of immunosuppression secondary to advanced cirrhosis, this infection progressed unchecked and ultimately led to death.

## Discussion

Cirrhosis is increasingly recognized as a state of immune dysfunction, rendering affected individuals more susceptible to infections. Lower respiratory tract infections, including pneumonia, represent the most lethal extra-peritoneal manifestations of bacterial infections in patients with cirrhosis, accounting for a significantly increased risk of mortality compared to other infectious complications [6].

Animal bite wounds are commonly associated with a polymicrobial flora, including aerobic organisms, such as *Staphylococcus* spp., *Streptococcus* spp., *Pasteurella* spp., *Capnocytophaga* spp., *Moraxella* spp., *Corynebacterium* spp., and *Neisseria* spp., as well as various anaerobic bacteria [7].

Among the most frequently implicated pathogens are *Pasteurella (P.) *species, with *Pasteurella multocida* being the predominant isolate [8]. Pasteurellosis may also be caused by four additional species: *P. septica*, *P. canis*, *P. stomatis*, and *P. dagmatis *[9]. *P. canis *was the predominant isolate identified in dog bite wounds, whereas *Pasteurella* subspecies multocida and septica were the primary isolates recovered from cat bite injuries [10]. A documented case has reported a dual wound infection caused by *P. dagmatis *and *P. canis* following a dog bite incident [11].

These gram-negative, facultatively anaerobic coccobacilli colonize the upper respiratory tract of many domestic animals, particularly cats and dogs. Cats are considered the primary reservoir, with higher carriage rates compared to dogs [12]. Localized infections caused by *P. multocida* typically manifest as acute cellulitis at the site of inoculation, often accompanied by erythema, swelling, and pain. In some cases, the infection may progress to abscess formation or deeper soft-tissue involvement [12]. Superficial soft tissue infections are most commonly reported in healthy individuals. However, *P. multocida*, acting as an opportunistic pathogen, can cause severe invasive infections in immunocompromised hosts [9,13]. Elderly patients with significant underlying comorbidities, particularly liver cirrhosis, chronic obstructive pulmonary disease (COPD), malignancies, or ischemic heart disease, are at increased risk of developing *P. multocida *bacteraemia, often following animal bites or scratches that facilitate bacterial entry [12].

*Bergeyella (B.) zoohelcum* is also an opportunistic zoonotic pathogen that has been increasingly recognized in infections following animal bites, particularly those inflicted by dogs or cats. Reported clinical manifestations include cellulitis, soft tissue abscesses, especially in the lower extremities--tenosynovitis, septicemia, pneumonia, and meningitis [14].

*Capnocytophaga (C.) *species are part of the normal oral flora of dogs and cats, with *C. canimorsus* and *C. cynodegmi* being the primary species implicated in human infections. These pleomorphic gram-negative rods are commonly transmitted through animal bites or through contamination of open wounds with animal saliva. While *C. canimorsus *is the species most frequently associated with severe systemic infections, *C. cynodegmi *typically causes more localized disease [15]. A *Capnocytophaga *spp. infection should be considered in the differential diagnosis when a patient develops multiorgan failure following a dog bite, particularly in the context of immunosuppression or delayed wound management [16].

*Neisseria* species have been recognized as potential pathogens in dog-bite-related infections since at least 1974. Among the species most frequently implicated are *Neisseria weaveri*, *Neisseria animaloris*, and *Neisseria zoodegmatis*. These organisms are considered part of the normal oral microbiota of dogs, cats, and, to a lesser extent, rodents. In human hosts, these zoonotic *Neisseria *species are most associated with purulent wound infections following animal bites. However, more invasive presentations have been reported in the literature, including pulmonary diseases, chronic otitis media, endophthalmitis, tenosynovitis, and bacteremia. These findings underscore the pathogenic potential of *Neisseria* spp. in both localized and systemic infections following zoonotic exposure [17].

According to the literature, most patients with *P. multocida* bacteraemia have underlying chronic illnesses, and those who succumb to the infection frequently suffer from severe immunosuppressive conditions. Liver cirrhosis, chronic renal failure, and malignancy are well-documented risk factors for opportunistic and invasive *P. multocida* infections. *C. canimorsus* is generally considered low-virulence; however, clinically significant infections typically occur in immunocompromised individuals. The majority of reported cases involved patients who had undergone splenectomy or alcoholism [16]. Notably, the mortality rate is elevated among patients who develop septic shock and multiorgan failure [18]. Although infections caused by *B. zoohelcum* are relatively rare, they may result in severe outcomes, particularly in individuals with predisposing conditions such as diabetes mellitus or cirrhosis. In such patients, the pathogen has been documented to cause systemic infections, including bacteremia. Therapeutically, *B. zoohelcum *demonstrates susceptibility to β-lactam antibiotics and fluoroquinolones, which are considered effective treatment options [14].

After soft tissue infections at the site of a bite, the respiratory tract represents the second most common site of *P. multocida* involvement [19]. Pulmonary manifestations may include pneumonia, tracheobronchitis, lung abscess, or empyema. Although the precise incidence of *P. multocida* infections remains unknown, largely due to the underdiagnosis of causative organisms in community-acquired pneumonia, the pathogen is underreported [12]. Respiratory tract infections associated with *Pasteurella* species typically occur in elderly individuals with chronic comorbidities, as observed in our case. Although rare, a variety of severe invasive diseases have been reported, including meningitis, endocarditis, and peritonitis [5,6].

Diagnosing *Pasteurella*-related respiratory infections can be clinically challenging, as their presentation can mimic that of more common respiratory pathogens. Therefore, prophylactic antibiotic therapy is recommended for high-risk populations. These include individuals with primary wound closure, edema, crush injuries, devitalized tissue, full-thickness wounds involving joints or tendons, puncture wounds, facial bites, and those who are immunocompromised or asplenic. Tetanus immunization is recommended following animal bites. When the patient’s immunization status is uncertain or incomplete, a full course of tetanus vaccination should be administered [1].

The antibiotic of choice for both prophylaxis and treatment of local *P. multocida* infections is oral amoxicillin-clavulanic acid, which is generally considered the first-line antibiotic for animal bites. In case of penicillin-resistant strains, doxycycline, trimethoprim/sulfamethoxazole, second-generation cephalosporins (e.g., cefuroxime), or fluoroquinolones (e.g., ciprofloxacin, levofloxacin) are recommended as an alternative therapy. A typical course of treatment lasts approximately 14 days [9,12,13]. From a microbiological standpoint, *C. canimorsus* is notable for its inability to produce β-lactamases, unlike other *Capnocytophaga *species. This characteristic renders it generally susceptible to β-lactam antibiotics, although it demonstrates intrinsic resistance to several antimicrobial classes, including aztreonam, trimethoprim-sulfamethoxazole, fosfomycin, and aminoglycosides [20].

A limitation of this report is that, as a postmortem forensic case, it does not allow direct correlation between clinical symptoms and disease progression in a living patient, which may limit its immediate applicability to routine clinical prediction. The lack of microbiological cultures and pathogen confirmation is another limitation of this case report, and further studies on microbiological involvement in such cases would be beneficial. Additionally, representative histological images of the lung were not available for inclusion, which limits the visual documentation of the pulmonary findings described. The lack of microbiological cultures and pathogen confirmation is another limitation of this case report, and further studies on microbiological involvement in such cases would be beneficial.

## Conclusions

This case underscores the forensic significance of zoonotic infections as a cause of death in vulnerable individuals, particularly those with underlying immunosuppressive conditions such as advanced cirrhosis. Given the immunological role of the splenic white pulp, its marked reduction likely contributed to immune dysfunction, thereby predisposing the individual to severe infection. The absence of medical intervention following a dog bite was associated with a neglected soft tissue infection, which subsequently progressed to a fatal lower respiratory tract infection.

The autopsy findings, including cellulitis of the upper limb and lobar pneumonia, support a plausible causal sequence linking the untreated dog bite to systemic infection in the context of advanced liver disease. From a forensic perspective, the manner of death is most appropriately classified as natural, with contributory external factors, namely, the dog bite, acting as the initiating event rather than the sole cause of death. This case highlights the importance of recognizing and documenting seemingly minor injuries during postmortem investigations, particularly when such injuries may precipitate fatal outcomes in medically compromised individuals.
